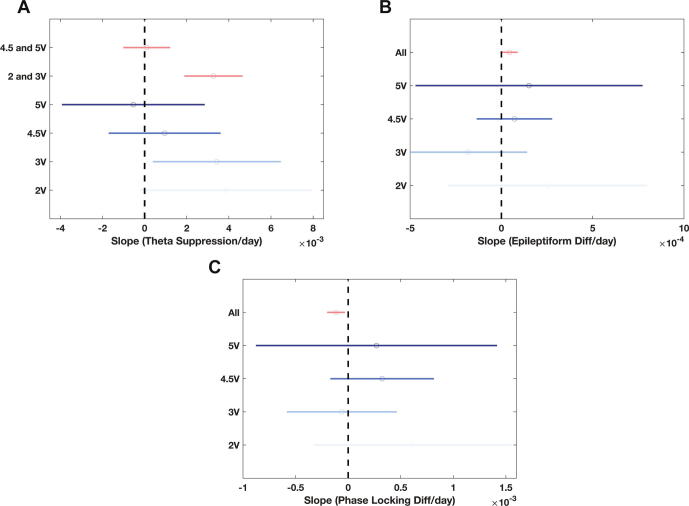# Effects of anterior thalamic nuclei stimulation on hippocampal activity: Chronic recording in a patient with drug-resistant focal epilepsy

**DOI:** 10.1016/j.ebr.2021.100467

**Published:** 2021-06-30

**Authors:** Alexander B. Silva, Ankit N. Khambhati, Benjamin A. Speidel, Edward F. Chang, Vikram R. Rao

**Affiliations:** a Medical Scientist Training Program, University of California, San Francisco, USA; b Department of Neurological Surgery and Weill Institute for Neurosciences, University of California, San Francisco, USA; c Department of Neurology and Weill Institute for Neurosciences, University of California, San Francisco, United States

**Keywords:** Deep brain stimulation, Chronic electrocorticography, Epilepsy, Hippocampus, Functional connectivity, Biomarkers of treatment

## Abstract

Implanted neurostimulation devices are gaining traction as palliative treatment options for certain forms of drug-resistant epilepsy, but clinical utility of these devices is hindered by incomplete mechanistic understanding of their therapeutic effects. Approved devices for anterior thalamic nuclei deep brain stimulation (ANT DBS) are thought to work at a network level, but limited sensing capability precludes characterization of neurophysiological effects outside the thalamus. Here, we describe a patient with drug-resistant temporal lobe epilepsy who was implanted with a responsive neurostimulation device (RNS System), involving hippocampal and ipsilateral temporal neocortical leads, and subsequently received ANT DBS. Over 1.5 years, RNS System electrocorticography enabled multiscale characterization of neurophysiological effects of thalamic stimulation. In brain regions sampled by the RNS System, ANT DBS produced acute, phasic, frequency-dependent responses, including suppression of hippocampal low frequency local field potentials. ANT DBS modulated functional connectivity between hippocampus and neocortex. Finally, ANT DBS progressively suppressed hippocampal epileptiform activity in relation to the extent of hippocampal theta suppression, which informs stimulation parameter selection for ANT DBS. Taken together, this unique clinical scenario, involving hippocampal recordings of unprecedented chronicity alongside ANT DBS, sheds light on the therapeutic mechanism of thalamic stimulation and highlights capabilities needed in next-generation devices.

## Highlights

• Devices for RNS and thalamic DBS were implanted in a single person with epilepsy.
• RNS electrocorticography enabled characterization of acute and chronic DBS effects.
• DBS caused acute, phasic, frequency-dependent responses in hippocampus and cortex.
• DBS modulated functional connectivity and suppressed epileptiform activity over time.
• Chronic electrocorticography elucidates progressive effects of thalamic stimulation.

## Introduction

Epilepsy, a debilitating neurological disorder characterized by recurrent seizures, affects over 50 million people worldwide [1] and has a lifetime prevalence of approximately 1 in 26 [2]. One-third of people with epilepsy suffer from seizures that are not controlled by medications, termed drug-resistant epilepsy. Many of these individuals are candidates for resection or ablation of seizure-producing brain tissue [3]. Although effective, these destructive, irreversible treatments may not be possible when seizures involve eloquent cortex or arise from multiple or spatially extensive foci. In these cases, several forms of brain electrical stimulation—including implanted devices for responsive neurostimulation (RNS) and deep brain stimulation (DBS)—represent palliative treatment options [4]. The most recently approved neurostimulation device for epilepsy delivers scheduled intermittent stimulation to the bilateral anterior thalamic nuclei (ANT DBS) and reduces median seizure frequency by up to 75% over several years, though efficacy is variable and seizure freedom is uncommon [5], [6], [7].

The modest efficacy of ANT DBS for reducing seizures may relate to precise anatomic targeting of stimulation [8] and to limited mechanistic understanding of this therapy. Indeed, there are no established neurophysiological biomarkers that clinicians can use to navigate a vast stimulation parameter space involving a myriad of potential combinations of electrodes, voltage, duty cycle, and frequency [9]. Device programming is thus necessarily based on experience from clinical trials, but this empiric approach may yield suboptimal therapeutic response. What is known about the neurophysiological effects of ANT DBS stems largely from animal studies along with short-term intracranial recordings in humans and hinges on connections between ANT, hippocampus, and neocortex in the circuit of Papez [10], [11]. For example, ANT stimulation modulates activity in the circuit of Papez [12] and suppresses local field potentials (LFPs) in the ipsilateral hippocampus [13], [14]. In sheep, the suppression of hippocampal LFP during ANT stimulation is specific for the theta frequency band [15]. Distributed effects of ANT stimulation have also been described, including desynchronization of large-scale brain networks [16]. Work in a semi-acute non-human primate model of seizures revealed that coherence-based functional connectivity between ANT and hippocampal activity predicts efficacy of ANT stimulation for short-term seizure reduction [17]. However, prior studies have not involved chronic *in vivo* neural recordings, limiting translation of these findings to human epilepsy where efficacy of ANT DBS improves gradually over several years of therapy [6], [7]. This limitation is particularly salient in light of an emerging conception of therapeutic neurostimulation that invokes progressive remodeling of the epileptogenic network over long periods of time with frequency-specific changes in LFP and regional connectivity [18], [19], [20]. However, direct evidence that ANT DBS promotes long-term remodeling of the epileptogenic network remains elusive, owing partly to the spatially limited sensing capability of ANT DBS technology [21].

Another FDA-approved neurostimulation device for epilepsy, the RNS® System, operates with a closed-loop design [22] and is one of only two commercial devices that stores a limited form of chronic intracranial electroencephalography (cEEG) [23]. RNS cEEG has been used to address challenges in clinical epilepsy—seizure lateralization [24] and localization [25], spell characterization [26], evaluating anti-seizure medications (ASMs) [27], and seizure forecasting [28]—and it has also proven to be a powerful tool for basic neuroscience research on cortical language representation [29], spatial memory [30], and other cognitive functions [31], [32]. RNS cEEG has helped characterize neural desynchronization related to vagus nerve stimulation [33] but, to our knowledge, it has not been used to quantify neurophysiological effects of an ANT DBS device implanted in the same individual.

Here, we leveraged a unique opportunity to study the long-term brain network effects of ANT DBS in a man with drug-resistant focal epilepsy who, for clinical indications, was treated concurrently with the RNS System connected to hippocampal and temporal neocortical leads. Our aim was to use hippocampal cEEG recordings made by the RNS System over a 1.5-year period to characterize acute and chronic neurophysiological effects of ANT DBS at different stimulation voltages and to relate these effects to changes in clinical and electrographic seizure frequency. We hypothesized that hippocampal responses to ANT DBS would be voltage-dependent and would correlate with the patient’s outcome.

## Methods

### Participant

Clinical outcome and device data were collected retrospectively, and study participation did not influence the patient’s clinical care. An electronic seizure diary was used to determine clinical seizure frequency. ASMs (phenytoin, levetiracetam) were not changed during the study period. This case report was exempted from Institutional Review Board approval at the University of California, San Francisco, but the participant provided express written consent for this study.

### Imaging and electrode localization

Prior to DBS and RNS implantation and after the resection of the right temporal lobe, 3 Tesla brain magnetic resonance imaging (MRI) was performed with acquisition of structural 3D T1 and T2 sequences and a diffusion tensor imaging (DTI) sequence consisting of 55 directions at 2000 s/mm^2 and one volume without diffusion restriction (GE Healthcare, Milwaukee, WI). T1 and T2 volumes were processed using FreeSurfer 6.0 for pial surface reconstruction [34]. DTI was corrected for distortions caused by eddy currents and susceptibility artifacts using fsl’s eddy, and outlier slices were replaced [35]. The corrected 4D volume underwent tensor reconstruction and whole brain tractography using DSI studio [36]. Fibers were filtered based on termination in thalamus, hippocampus, and inferior temporal gyrus of the left hemisphere based on the automated anatomical labeling (AAL) brain atlas [37]. DTI volume was co-registered to MRI in tkRAS coordinate space, and the transformation was applied to the fibers for display with the pial surface reconstruction. Post-operative head computed tomography (CT) acquired after the implantation of the RNS and DBS devices was co-registered to the 3D T1 MRI in tkRAS space. Coordinates of each electrode were recorded and displayed with the pial surface and tractography to localize each device relative to nearby anatomical structures.

### Device settings and clinical variables

RNS System comprised a cranially-implanted neurostimulator (model RNS-320) connected to a four-contact depth lead (1-cm contact spacing) placed trans-occipitally along the long axis of the left hippocampus and to a four-contact cortical strip lead (1-cm contact spacing) placed on the left inferior temporal gyrus (ITG). RNS System detection settings were constant throughout the study period (bandpass detectors applied to bipolar montage of the distal pair of hippocampal electrodes with the following parameters: 17–125 Hz, 4% amplitude, 0.512 s; 2–125 Hz, 32% amplitude, 0.512 s) and ‘Long Episodes’ (detections of abnormal activity sustained over 30 s) stored by the device were a reliable proxy for electrographic seizures (>90% positive predictive value, as determined by methods described previously [28], [38], [39]). RNS System electrocorticograms (ECoGs) are 4-channel bipolar-montaged recordings sampled at 250 Hz and stored in response to triggers that include Long Episodes and predetermined times of day (‘Scheduled’ recordings). The RNS System stimulation pathway was monopolar and involved the left hippocampal depth lead only, due to clear seizure onset from the hippocampus before spread to the ITG. RNS System stimulation parameters, held constant during the study period, were: current 4.5 mA, frequency 333 Hz, pulse width 160 µs, burst duration 100 ms. ANT DBS involved a chest implanted pulse generator (Medtronic Activa^TM^ PC) connected to two four-contact depth leads (Model 3389, 0.5-mm contact spacing) placed via trans-ventricular approach into bilateral ANT. The second most proximal electrode contact on each lead was designated as the cathode, with frequency 145 Hz, pulse width 90 µs, and stimulation-cycling mode of 1-min on and 5-min off. Of the tunable device parameters, only DBS stimulation voltage was periodically adjusted (range 2–5 V) based on clinical factors. The RNS System is electrically isolated from the ANT DBS device, mitigating the confounding effect of electrical noise and saturation of the recording amplifier during stimulation delivery [40].

### Data collection and Pre-processing

We analyzed cEEG recordings from the RNS System over approximately 1.5 y of ANT DBS therapy. Two types of RNS ECoGs, set at 180 s duration in this individual, were used: ‘Scheduled’ recordings, stored at four pre-defined times of day (00:00, 06:00, 12:00, 18:00); and ‘User Saved’ recordings, longer (up to 15 min) segments of real-time ECoG streamed by wand telemetry during clinic visits. Given that the RNS System and ANT DBS devices operate independently, DBS ‘on’ intervals could fall at the beginning, middle, or end of a Scheduled RNS ECoG recording (Fig. S2A). We analyzed the DBS stim-on interval present in each recording and comparing it to a duration-matched DBS stim-off interval from the same recording (Fig. S2B).

**Fig. 1:**
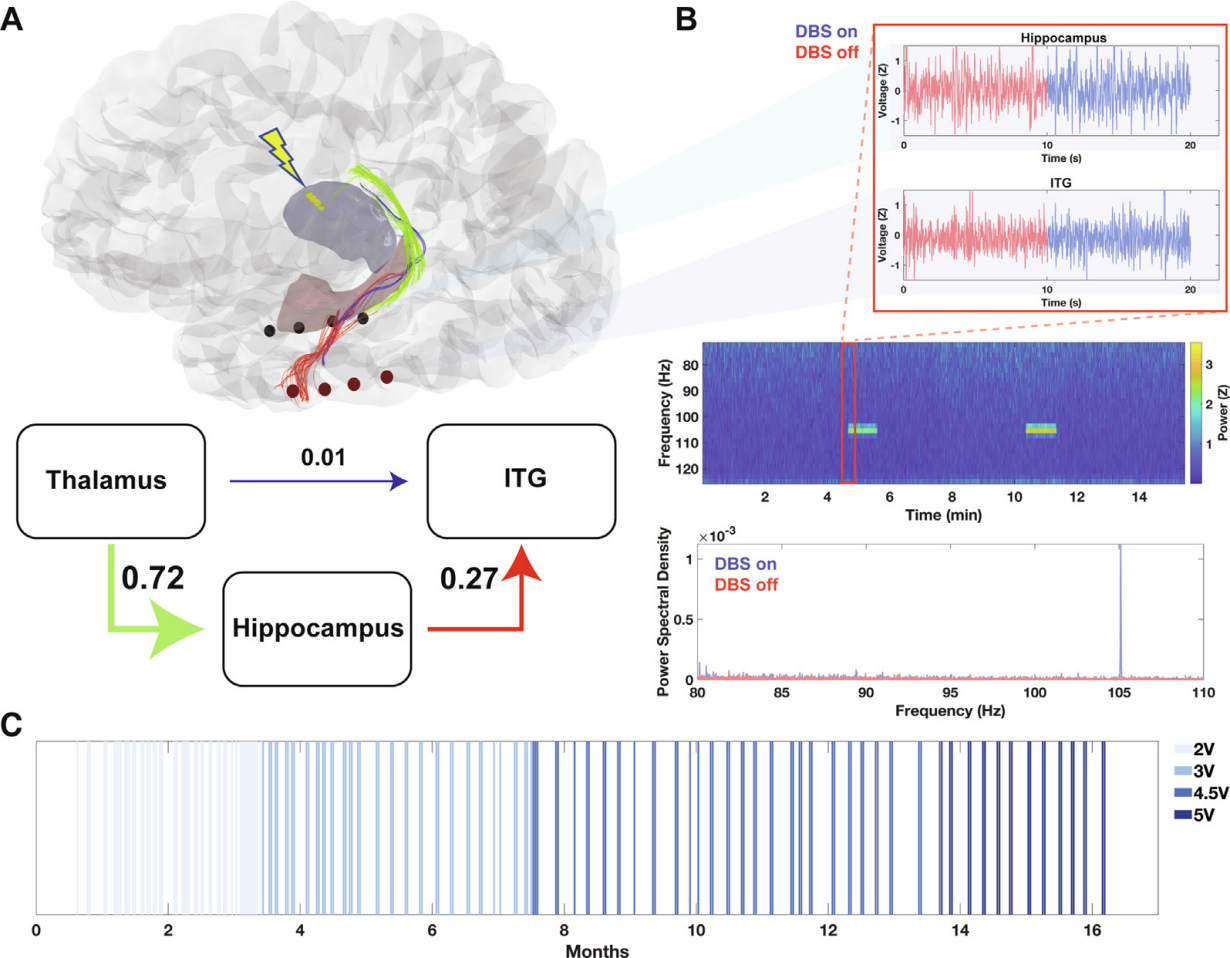
ANT DBS and RNS System in one individual. **(A)** Top, reconstruction of DBS electrodes (yellow contacts) and RNS electrodes (red and black contacts). Tractography streamlines connecting the thalamus to hippocampus (green), thalamus to ITG (blue), and hippocampus to ITG (red) are overlaid. Bottom, weighted structural connectivity map, where edge weight represents normalized streamline count between regions sampled by electrodes. **(B)** Spectrogram of RNS ECoG from a hippocampal electrode pair streamed over 15 minutes. DBS stimulation artifact occurs for 1 min every 5 min, consistent with ANT DBS duty cycle. Inset, raw RNS ECoG from both the hippocampus and ITG during ANT DBS on (artifact) and off intervals showing no obvious differences in the raw ECoG signal. Power spectral density plot during ANT DBS on vs. off intervals localizes artifact to 105 Hz, as predicted based on stimulation frequency of 145 Hz and RNS System Nyquist limit of 125 Hz. **(C)** Raster plot of > 300 RNS ECoGs analyzed in this study color-coded by ANT DBS voltage at each time. X-axis represents months post implantation with DBS device.

A bipolar montage and 4–90 Hz bandpass filter are applied on the RNS System neurostimulator [30] and the amplifier is turned off during delivery of stimulation, so RNS System stimulations are evident by a brief (typically 40 ms) blanking of the ECoG signal. We leveraged these blanking periods to detect RNS stimulation events and to remove corresponding time segments from our analysis. Notch-filtering at 105 Hz removed volume conduction artifact of DBS (Fig. 1B, and see below). Each Scheduled recording waveform was standardized by a Z-score transformation.

### Determining DBS on/off periods

**Supplementary Fig. 1:**
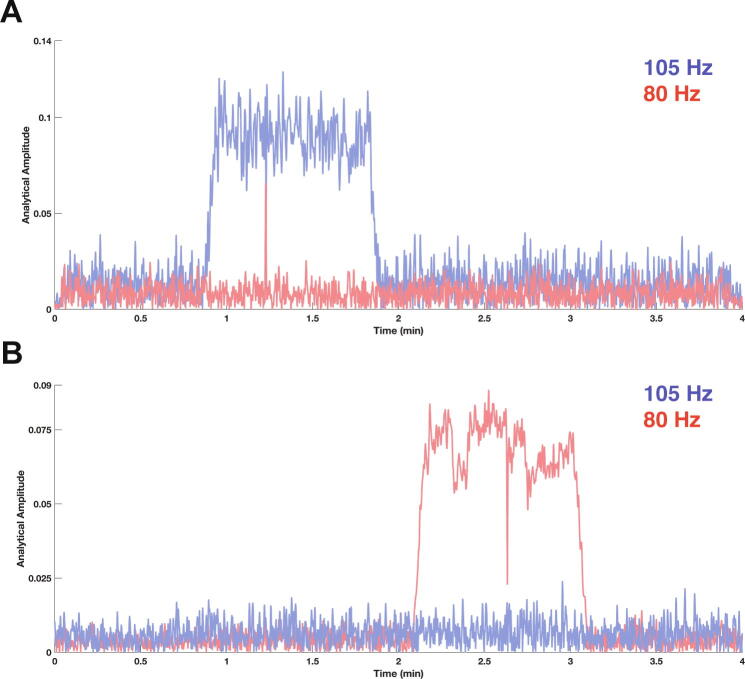


A 105 Hz artifact was present in ECoG spectrograms across all electrodes, occurring for 1 minute every 5 minutes (Fig. 1B). This artifact may be explained by volume conduction of a DBS frequency of 145 Hz, which is 20 Hz over the Nyquist rate of the ECoG signal (125 Hz) and is therefore mapped to 20 Hz below the Nyquist rate. We validated use of this artifact to define DBS active intervals in two ways: First, we temporarily disabled ipsilateral (left) thalamic stimulation, leaving contralateral stimulation on, and confirmed persistence of the artifact, suggesting volume conduction rather than neural propagation (Fig. S1A). Second, we briefly changed stimulation frequency to 80 Hz and confirmed corresponding shift of the artifact from 105 to 80 Hz (Fig. S1B). To determine when DBS was active during 180 s Scheduled RNS ECoG recordings, we extracted the 105 Hz analytical amplitude using the real component of the Hilbert transform. We then Z-scored the analytical amplitude and searched for consecutive time intervals longer than 10 seconds when the 105 Hz amplitude was greater than 0. These intervals constituting “DBS on” times were checked manually to ensure accurate classification.

### Frequency band specific LFP analysis

We analyzed ECoG signals in canonical frequency bands (delta: 1–5 Hz, theta: 5–10 Hz, alpha: 10–15 Hz, beta: 15–25 Hz, low-gamma: 30–40 Hz, and high-gamma: 70–90 Hz [41], [42]) extracted by bandpass filtering the pre-processed ECoG waveform. The real component of the Hilbert transform gave the analytic amplitude of each band as a time series. Analytic amplitude for each band was Z-scored using the time interval of −20 to −10 seconds (0 is onset of DBS) as a reference. Statistical testing for a given electrode and frequency band was performed using a paired t-test to compare the mean analytical amplitude during the time interval −10 to 0 and running intervals of 5 seconds post DBS on in each middle-scheduled recording. To correct for multiple comparisons, we used Bonferroni-corrected adjusted alpha level of 0.05/n, where n is the number of tests (n = 14, alpha = 0.0036).

### Computing measures of functional connectivity

To measure functional connectivity, we computed phase-locking and amplitude correlation between ECoG waveforms in the hippocampus and ITG. We analyzed the frequency bands that were found to be significantly modulated by DBS: theta, beta, and high gamma. For each Scheduled ECoG that contained a DBS ‘on’ interval, we randomly selected a time-matched interval during the recording when DBS was off. We then compared each functional connectivity metric between intervals for a given Scheduled ECoG. Amplitude coherence was computed by taking the correlation between the raw real Hilbert transformed time series for frequency bands of interest and electrodes. Phase-locking coherence was similarly computed based on the raw real Hilbert transformed time series. To statistically compare DBS on vs. off intervals, a paired non-parametric rank sum test was used with an adjusted alpha level of 0.05/n where n is the number of electrode permutations (n = 4, alpha = 0.0125).

### Quantifying clinical effects

Rate of epileptiform activity was defined as the count of RNS detections [38] divided by the length of the time interval. It was compared between DBS on and off intervals using a non-parametric rank sum test. All electrographic seizure frequency data provided in the study corresponds to Long Episodes, a reliable proxy for electrographic seizures in the participant. To quantify the relationship between the magnitude of theta suppression and DBS voltage, we calculated the mean LFP difference between the 5 s interval preceding DBS on and the 5 s interval directly following DBS on for each band. We then used a one-way ANOVA followed by post-hoc Tukey-Kramer tests, at an alpha of 0.05, to probe differences between stimulation voltage settings. To quantify network and LFP effects over time, we used ordinary least squares linear regression.

## Results

### Case history

A 44-year old right-handed man presented with drug-resistant focal epilepsy that began at age 28. He had focal impaired awareness seizures involving déjà vu and right upper extremity automatisms. Pre-surgical evaluation, including intracranial EEG monitoring, localized seizures to the right temporal lobe. He underwent right anterior temporal lobectomy followed, due to persistence of seizures, by posterior extension of the resection two years later. Seizures recurred within the first month post-operatively, and scalp-based video-EEG monitoring seizures that now had onset over the left temporal lobe with a new semiology, involving left upper extremity automatisms, right upper extremity dystonic posturing, speech arrest, and right head version. Resection of the left temporal lobe was precluded by prior contralateral temporal resections, so the RNS System was implanted with a left trans-occipital hippocampal depth lead and a cortical strip lead over the left lateral temporal neocortex (inferior temporal gyrus, ITG). Over the next 3.5 years, RNS System therapy resulted in significant reduction in clinical seizure frequency, from a pre-RNS baseline of one seizure per day to one seizure per month. However, the patient desired seizure freedom and requested additional treatments, leading to implantation of an ANT DBS device. Following DBS implant, RNS System detection and stimulation settings were held constant and only the DBS stimulation voltage was adjusted periodically, about every 3 months, over 1.5 years. During that time, the RNS System provided cEEG data that supplemented patient-reported seizures [43], including counts of detected epileptiform discharges and electrographic seizures (see Section 2.8.).

### Thalamo-cortical structural connectivity

DBS electrodes were mapped to the patient’s T1-weighted MRI and localized to the ANT bilaterally. RNS System electrodes localized to the left hippocampus and ITG, as expected (Fig. 1A). DTI-based fiber tracking revealed evidence of robust structural connectivity between the thalamus and hippocampus and between the hippocampus and ITG. Comparatively fewer streamlines were observed between the thalamus and ITG, suggesting a putative route for thalamic stimulation to propagate from the ANT to the hippocampus and from there to the ITG (Fig. 1A).

### Aligning ANT DBS with RNS ECoGs

The RNS and DBS neurostimulators do not interact directly, so we sought to develop methods to align thalamic stimulation on/off times in RNS ECoGs. Visually, there was no obvious difference in the raw RNS ECoG between DBS on and off periods (Fig. 1B, inset). With spectral analysis, however, high-frequency DBS stimulation artifact was evident in RNS ECoGs, enabling segmentation of thalamic stimulation on vs. off intervals (Fig. 1B; see Section 2.5.). This artifact was specific to the simulation frequency of DBS (Fig. S1) and was concordant with DBS duty cycle (Fig. 1B). We collected and analyzed over 300 RNS ECoGs between May 2019 and October 2020. The only ANT DBS parameter that varied during this time was stimulation voltage (Fig. 1C).

### Acute thalamic stimulation effects on hippocampal and cortical activity

**Fig. 2:**
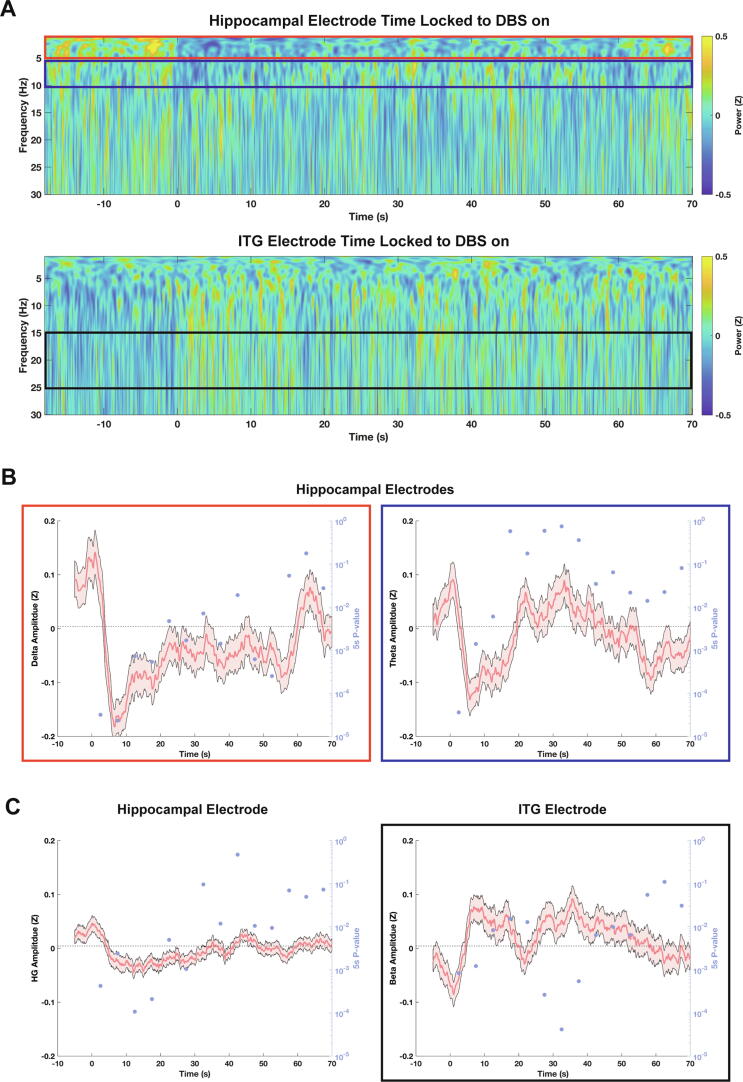
Acute, frequency-dependent effects of ANT DBS. **(A)** Low frequency spectrograms, averaged across all ‘Middle’ Scheduled RNS ECoGs, for a hippocampal and an ITG recording channel. ANT DBS is on between time 0 and 60. **(B)** Quantification of analytical amplitude in the delta and theta bands for a hippocampal recording channel, time-locked to the onset of ANT DBS (time 0). **(C)** Quantification of other frequency bands that responded during ANT DBS. Left, high gamma amplitude in the hippocampal channel from (B); Right, beta amplitude for an ITG channel. Error bars represent standard error of the mean. P-values are calculated using a paired t-test to compare the mean of each trial in the baseline interval (-10 to 0 seconds) to a 5-second test interval post-DBS onset. Horizontal black line indicates threshold for statistical significance based on comparison to adjusted alpha of 0.05 divided by the number of time intervals tested.

**Supplementary Fig. 2:**
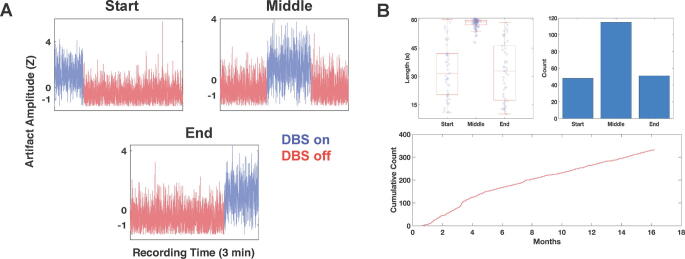


Leveraging the unique opportunity to sense mesial temporal and temporal neocortical activity during delivery of thalamic stimulation, we probed the network dynamics of ANT DBS on LFPs as recorded by RNS hippocampal and ITG electrodes. Based on structural connectivity analysis (Fig. 1A), we expected that the neurophysiological effects of ANT DBS would be stronger in the hippocampus than in the ITG. To test this, we aligned all ‘middle’ scheduled ECoGs (Fig. S2) by the beginning of DBS stim-on periods and then averaged time-locked spectrograms to visualize changes in ECoG frequency composition associated with the onset of stimulation (Fig. 2A).

We first visualized the analytic amplitude over time in canonical low frequency bands, delta (1–5 Hz) and theta (5–10 Hz). In line with previous studies [15], onset of thalamic stimulation was associated with suppression in hippocampal low frequency LFPs (Fig. 2A). By comparing consecutive 5 s intervals after DBS stim-on to the 10 s before stimulation, we found that low-frequency LFP suppression was transient (Paired student’s t-test with adjusted alpha of 0.05 divided by the number of time intervals tested: p < 0.05/14 = 0.0036). Specifically, theta band power demonstrated phasic reduction and statistically returned to baseline by 10 s, even though stimulation persisted for 60 s (Fig. 2B). Delta band power demonstrated strongest phasic reduction in the first 10 s of stimulation but remined reduced up to 60 s (Fig. 2B). Next, we examined higher frequency bands: alpha, beta, low gamma, and high gamma. We found that hippocampal high gamma activity was suppressed (Fig. 2C) but that ITG beta (15–25 Hz) activity was activated during DBS (Fig. 2A, C). Again, these effects were transient during DBS stim-on periods. Thus, DBS produces acute, transient suppression of hippocampal theta, delta, and high gamma activity with concurrent activation of ITG beta.

### Network-level effects of DBS: Specific modulation of phase-locking

**Fig. 3:**
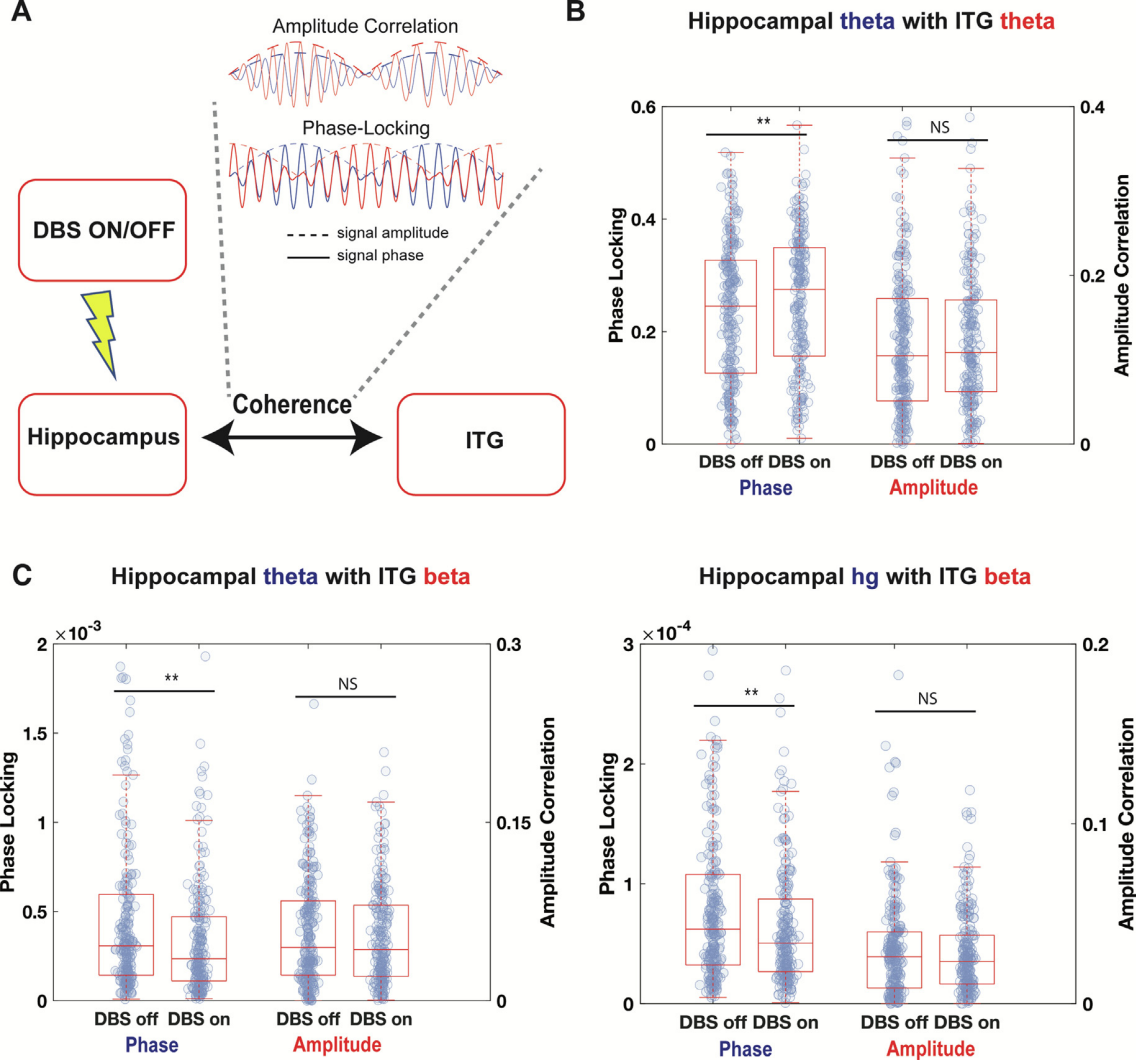
Effects of ANT DBS on functional connectivity. **(A)** Functional connectivity analysis scheme. Phase-locking and amplitude correlation between hippocampal and ITG electrodes were calculated for frequency bands that responded to stimulation (Fig. 2). **(B-D)** Pairwise Phase-locking and amplitude correlation between hippocampal and ITG activity in specific frequency bands as indicated. Statistics for **B, C,** and **D** were based on non-parametric paired rank sum tests (significance based on comparison to 0.05/n where n was the number of tests). Box plots consist of the median with 25th and 75th percentiles and whiskers extending to the most extreme non-outlier point.

We next examined whether ANT DBS modulates synchronized neural interactions, or functional connectivity, between the hippocampus and temporal neocortex. Given our structural connectivity results (Fig. 1A), we hypothesized that modulation of hippocampal neural activity by ANT DBS would, in turn, synchronize neural activity in ITG. To test this, we measured functional connectivity between hippocampal and ITG electrodes using the phase-locking coherence and amplitude coherence metrics (Fig. 3A). Based on our earlier findings of acute, transient effects of DBS, we focused our functional connectivity analysis on the theta, beta, and high gamma bands. For a sample pair of electrodes, we found that hippocampal and ITG theta had a higher level of phase-locking during stimulation (Fig. 3B; Wilcoxon signed rank test: p = 0.0051). Hippocampal to cortical cross-frequency phase-locking promotes neural plasticity and underlies memory processes [44], so we asked whether DBS modulated the cross-frequency phase-locking of hippocampal theta and high gamma to ITG beta. We found that during DBS stimulation such cross-frequency phase-locking interactions are decreased (Fig. 3C; Wilcoxon signed rank test: p = 0.00058 and 0.00061)). Amplitude correlation showed no significant differences during stimulation (Fig. 3B, C). Thus, the phase-locking of theta oscillations between the hippocampus and ITG is increased by DBS, whereas phase-locking of hippocampal theta and high gamma oscillations with respect to ITG beta decreases during DBS. More generally, our findings demonstrate that ANT DBS is more likely to modulate phase-based interactions between hippocampus and ITG than amplitude-based interactions. To investigate how this network-level modulation of brain activity might support therapeutic suppression of epileptic brain activity, we next turned our attention to examining DBS effects on interictal epileptiform discharges and seizures.

### Chronic effect of ANT DBS on interictal epileptiform activity

**Fig. 4:**
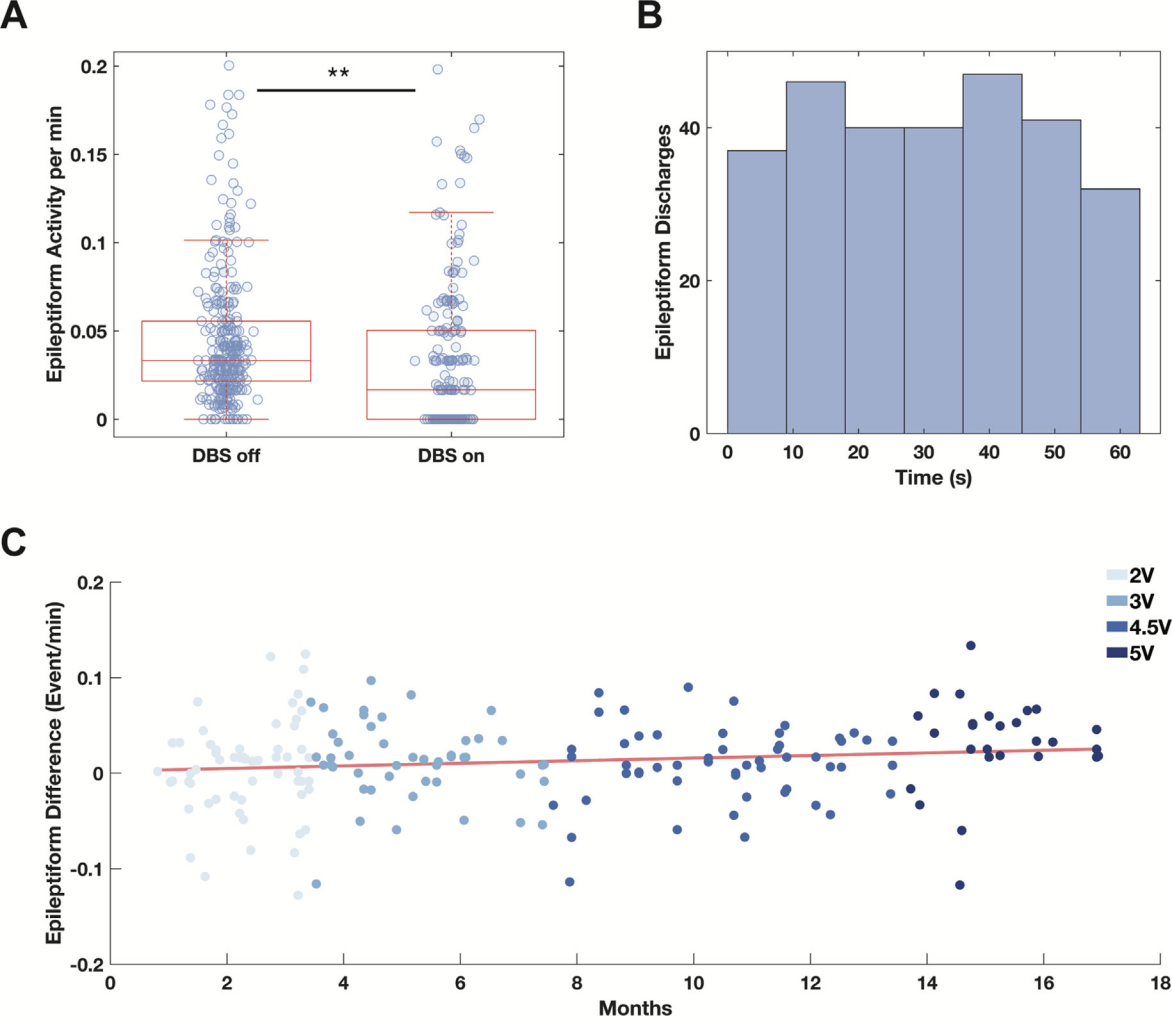
Effects of ANT DBS on epileptiform activity. **(A)** Fewer RNS System detections of epileptiform activity occur during ANT DBS stim-on intervals than during stim-off intervals (P < 0.001, rank sum test). **(B)** Suppression of epileptiform activity persists throughout ANT DBS stim-on periods. Histogram of epileptiform activity detections in 10-s bins across 60-s stimulation periods. **(C)** Suppression of epileptiform activity by ANT DBS increases slowly over time (linear regression: slope = 4.46 × 10^-5^ difference/day, p-value = 0.052, r = 0.14). Pairwise epileptiform difference is defined as the difference in rate of epileptiform activity (events/min) between DBS off and on intervals for each scheduled ECoG.

**Supplementary Fig. 3:**
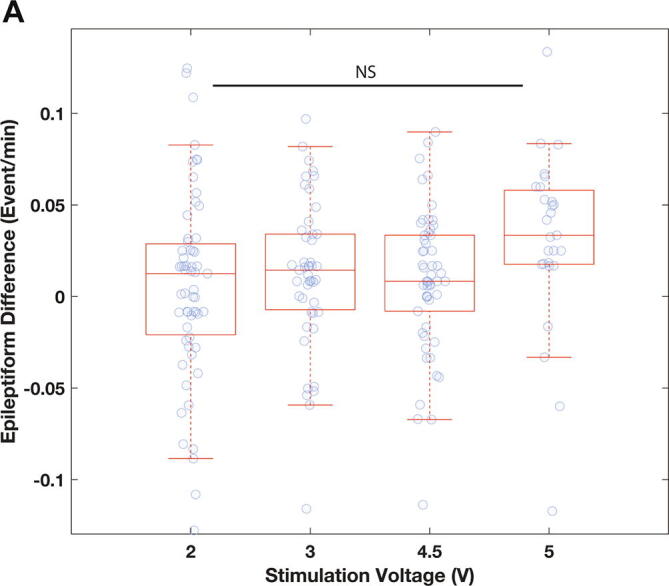


For normal clinical use of the RNS System, customizable, patient-specific algorithms are programmed on the neurostimulator to enable detections of epileptiform activity, and counts of these detections are an established proxy for interictal epileptiform discharges [28], [38], [39], [45]. During DBS stim-on intervals, the rate of RNS detections was significantly lower than during DBS stim-off intervals (Fig. 4A; rank sum: p < 0.001). In contrast to the transient suppression of LFP during ANT DBS (Fig. 2A), the effect of stimulation on the rate of detections was uniform across the entire 60 s of stimulation (Fig. 4B; Kolmogorov Smirnov test against uniform distribution: p > 0.05) and was not voltage-dependent (Fig. S3; ANOVA: p > 0.05). Additionally, we leveraged the chronicity of RNS recordings to visualize how the extent of epileptiform activity suppression (epileptiform difference) changed over time. We found that the level of suppression was relatively stable but increased slowly over time (Fig. 4C; linear regression: slope = 4.46 × 10^-5^ difference/day, p = 0.052, r = 0.14). Individual regression slopes for each voltage were not different from each other with confidence intervals including zero (Fig. S4A).

### Acute stimulation response as a biomarker of treatment efficacy

**Fig. 5:**
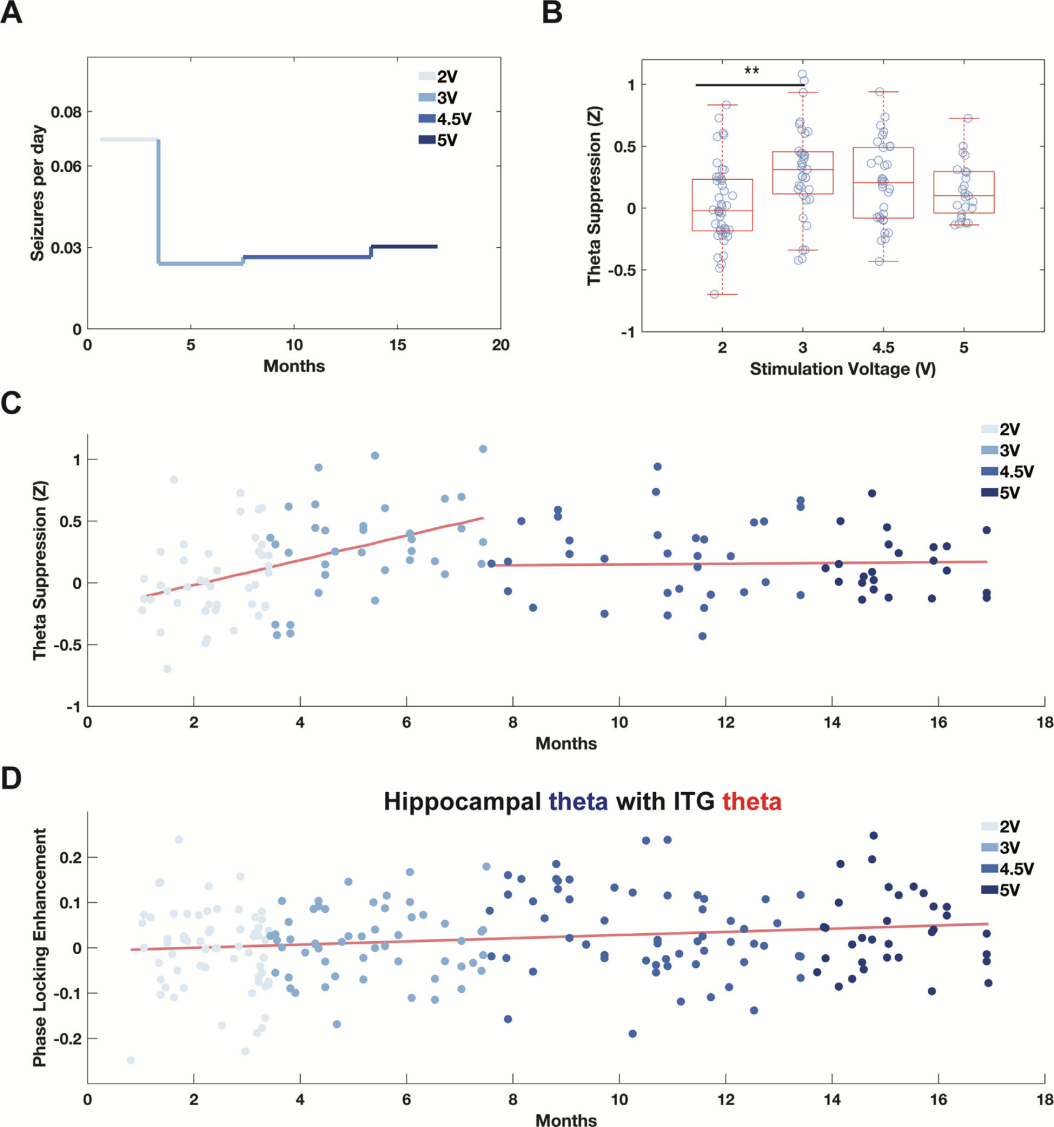
Neurophysiological correlates of seizure frequency reduction. **(A)** Mean electrographic seizure frequency at each ANT DBS stimulation voltage over time. **(B)** ANT DBS-induced hippocampal theta suppression at each stimulation voltage (P < 0.01, ANOVA with post-hoc Tukey Kramer tests) and **(C)** as a function of time: 2 and 3 V (linear regression: slope = 0.0033 Z/day, p-value = 1.5 × 10^-5^, r = 0.47) and 4.5 and 5 V (linear regression: slope = 1.0318 × 10^-4^ Z/day, p-value = 0.86, r = 0.0245). **(D)** Temporal dynamics of hippocampal and ITG theta phase-locking enhancement (Fig. 3B) during ANT DBS (linear regression: slope = 1.15 × 10^-4^ /day, p-value = 0.0082, r = 0.18).

We next studied the relationships between cumulative therapy, stimulation voltage, and clinical outcome by quantifying the acute effect of stimulation on LFP and functional connectivity. Using counts of Long Episodes (see Section 2.8.), we found electrographic seizure frequency decreased when ANT DBS was increased from 2 V to 3 V and remained low at 4.5 and 5 V (Fig. 5A). Given the critical role of hippocampal theta oscillations in many forms of cognition and behavior [46], we next asked whether acute suppression of the hippocampal theta rhythm during stimulation could assay chronic change in electrographic seizure frequency. We measured hippocampal theta suppression as the degree reduction in theta power during DBS on (see Section 2.8.). We found that theta suppression varied non-linearly with voltage and was greatest at 3 V (Fig. 5B). Additionally, we quantified how theta suppression evolved chronically over therapy (Fig. 5C) and found a significant linear relationship for 2 and 3 V (Linear regression: slope = 0.0033 Z/day, p = 1.5 × 10^-5^, r = 0.47); however, for the settings of 4.5 and 5 V, there was no significant linear relationship (linear regression: slope = 1.0318 × 10^-4^ Z/day, p = 0.86, r = 0.0245). We separately investigated the Individual regression slopes for theta suppression vs. time for each voltage setting (Fig. S4B). Next, we analyzed the temporal dynamics of the pairwise theta phase-locking enhancement (DBS on − control windows) between the hippocampus and ITG (Fig. 5D). We found that the enhancement of functional connectivity during stimulation increased over time (linear regression: slope = 1.15 × 10^-4^ /day, p = 0.0082, r = 0.18). This effect was not voltage-dependent, supported by overlapping confidence intervals of slope within each voltage setting (Fig. S4C). Overall, in this patient, seizure frequency reduction was correlated with an increase in hippocampal theta suppression, which linearly increased during the first two voltage settings. Functional connectivity enhancement during DBS increased throughout treatment, irrespective of voltage. Additionally, as stimulation voltage was increased further (4.5 and 5 V) to try and achieve greater efficacy, the patient developed a tremor in his right hand and reported an increase in clinical seizure frequency, though this was not reflected in electrographic seizure counts.

## Discussion

Here, we describe a novel system for monitoring and mapping the acute and chronic neurophysiological effects of ANT DBS. Our system entails implantation of two devices in a single patient—one device delivers open-loop electrical stimulation to the ANT and another device provides cEEG and an ability to quantify epileptiform activity over long periods of time. Using this composite system, we found that ANT DBS acutely modulates hippocampal activity in a voltage- and frequency-dependent manner, likely through direct anatomic connections between these structures. ANT DBS also modulates frequency-specific coherence between hippocampus and temporal neocortical regions, illustrating in principle how stimulation of deep nuclei can have effects that reverberate through large-scale brain networks. In concordance with prior work [16], we found that ANT DBS suppresses hippocampal epileptiform activity during stimulation, an effect that was not dependent on the stimulation voltage and that remained stable over 1.5 years. When hippocampal theta suppression was highest (stimulation voltage of 3 V), seizure frequency was lowest. Finally, theta phase-locking enhancement between the hippocampus and ITG during stimulation increases over time in a linear manner independent of stimulation voltage. Taken together, these results characterize voltage- and time-dependent effects of ANT DBS relative to unprecedented chronicity.

Our study makes several contributions to the existing literature. First, the neurophysiological effects of ANT DBS may be broadcast to brain networks extending beyond putative seizure foci. Second, our work showcases the utility of a dual-device system to chronically monitor stimulation-evoked human brain activity in cortical and subcortical brain regions. The chronicity of these effects helps elucidate gradual wash-in, wash-out, and adaptation kinetics of electrophysiological biomarkers that are otherwise difficult to discern during acute, in-hospital intracranial EEG monitoring. We extend previous work [16] showing an immediate and continuous suppression of interictal epileptiform discharges with thalamic stimulation during inpatient monitoring by demonstrating that the acute suppressive effects of stimulation on interictal epileptiform activity gradually strengthen over ANT DBS therapy. Third, our analyses support the emerging perspective that neurostimulation operates over divergent timescales—acute, subacute, and chronic—to achieve therapeutic effects in human epilepsy. Indeed, acute effects of ANT DBS persist beyond the cessation of individual stimulation events and accumulate over the duration of therapy.

In clinical practice, a major factor limiting the use of neurostimulation devices involves the ‘black box’ nature of the therapy and the current inability to predict effects of stimulation settings on neural activity. Our study suggests that putative neurophysiological biomarkers, such as acute suppression of hippocampal theta and delta band power, may help objectively discern whether thalamic stimulation has effectively reached more distant, downstream targets and consequently guide selection of device settings from a vast parameter space. If network-based biomarkers are validated in cEEG recordings, then they can potentially be probed via stereo-EEG [47] prior to implantation of indwelling hardware, facilitating identification of optimal candidates. Thus, our study extends the utility of cEEG and motivates development of next-generation devices [48] that can better provide this information.

Our findings also shed light on fundamental mechanisms underlying the acute and chronic neurophysiological effects of ANT DBS. First, targeted neurostimulation can modulate brain activity beyond primary endpoints through a cascade of downstream anatomic connections, raising the possibility that thalamic stimulation can penetrate more difficult-to-access areas of the brain. Multi-modal imaging techniques that merge empirical data from chronic ECoG with existing graph theoretic models of stimulation flow through an anatomic network [49] may help generate better predictions of the potential pathways of stimulation for individual patients. Second, thalamic DBS may not only modulate activity of multiple brain areas but also disrupt or enhance functional connectivity between them. Understanding the degree to which the spatially distributed effects of ANT DBS modulate functional connectivity associated with cognitive processes, like memory, is critical for minimizing side effects and making therapy safer. Third, we demonstrate divergent effects of thalamic stimulation on spectral activity, which exhibits phasic suppression at least an order of magnitude shorter than the typical duration of a single stimulation train, and on interictal epileptiform activity, which exhibits sustained suppression during a single stimulation train. Indeed, phasic changes in spectral activity occur at the onset of stimulation and may reflect the beginning of a more complex cascade of neurophysiological changes leading to more sustained suppression of epileptiform activity during stimulation delivery. In light of these dynamics, it may be possible to tune the duty cycle of ANT DBS stimulation to trigger reliably both a cascade of events to intermittently suppress epileptiform activity and also minimize duration of stimulation trains to maximize device battery life.

Finally, by leveraging chronicity of ECoG data over 1.5 years, we provide novel insight into the temporal dynamics of both hippocampal LFP and distributed network effects of ANT DBS. Theta suppression increased linearly throughout the first half year of treatment when stimulation voltage was kept at 2 and 3 V. This demonstrates that ANT DBS modulation of hippocampal LFPs is not immediate; rather, it develops over a sensitization period. Additionally, when the stimulation voltage was increased to 4.5 V, theta suppression remained at relatively constant levels. Thus, the desired neurophysiological effects are not instantaneous and are not necessarily enhanced by increases in stimulation voltage. Given our patient’s increased side effects at high voltage, such as tremor and difficulty sleeping, we suggest that swift increases in stimulation voltage may be counterproductive.

Our study has limitations. First, this a case report of one patient, so findings should be regarded as hypothesis-generating for future studies on larger datasets. Second, our analysis was retrospective, so our findings are correlative, and causality cannot be inferred. Despite these constraints, medication and stimulation/detection settings on the RNS System were held constant over the study period, and stimulation voltage was the only ANT DBS parameter that was periodically adjusted. Furthermore, each ‘Long Episode’ event detected by the RNS System reflected a separate occurrence of an electrographic seizure, providing unprecedented resolution into fluctuation in seizure rate alongside chronic therapy. Nonetheless, we cannot discount the possibility that the combination of ANT DBS, RNS, and ASMs synergistically drive chronic change in seizure occurrence. Third, the RNS System has limited spatial sampling and our analysis was limited to ECoG recordings from two brain regions. Furthermore, the RNS System is not capable of recording continuous ECoG, which necessitated analysis of ECoG recordings with truncated DBS stim-on periods. Fourth, this patient underwent prior resective surgery of the contralateral temporal lobe, which could theoretically alter the pathophysiology of brain networks involved in epilepsy and produce atypical neural responses to thalamic stimulation. However, about 25% patients studied in the pivotal SANTE (Stimulation of the Anterior Nucleus of the Thalamus) clinical trial [5] had undergone previous epilepsy surgery.

Our novel dual-device system inspires several future research directions. The interplay between multiple implanted devices distributed at key sensing and stimulation hubs throughout a patient’s brain network may eventually yield the capability to precisely drive brain network dynamics from an unhealthy, epileptogenic state to a healthy state. This study presents a proof-of-concept design for such a next-generation system [48], which should be equipped with onboard processing to monitor short-term and long-term network-based biomarkers of clinical response and optimize stimulation parameters and electrode configurations adaptively to deliver safer and more effective therapy. In the future, separate devices that have distinct, specialized functions for therapy delivery and neurodiagnostics should interact synergistically or be consolidated within a single device [50] that could help further demystify the ‘black box’ nature of neurostimulation for epilepsy.

**Data and code accessibility**: Code used in this study will be available upon publication at: https://github.com/akhambhati/zappy/blob/master/zappy/sigproc/connectivity.py.

## Disclosures

V.R.R. has served as a consultant for NeuroPace, Inc., manufacturer of the RNS System, but declares no targeted funding for this work. Other authors declare no conflicts of interest.

## Ethical Statement

We confirm that we have read the Journal’s position on issues involved in ethical publication and affirm that this report is consistent with those guidelines.

## Declaration of Competing Interest

The authors declare that they have no known competing financial interests or personal relationships that could have appeared to influence the work reported in this paper.

## Figures and Tables

**Supplementary Fig. 4:**